# Changes in hormonal profiles during competition preparation in physique athletes

**DOI:** 10.1007/s00421-024-05606-z

**Published:** 2024-09-11

**Authors:** Ville Isola, Juha J. Hulmi, Theo Mbay, Heikki Kyröläinen, Keijo Häkkinen, Vilho Ahola, Eric R. Helms, Juha P. Ahtiainen

**Affiliations:** 1 Faculty of Sport and Health Sciences, Neuromuscular Research Center, University of Jyväskylä, P.O. Box 35, 40014 Jyväskylä, Finland; 2 Institute of Public Health and Clinical Nutrition, University of Eastern Finland, 70211 Kuopio, Finland; 3 Sports and Exercise Medicine, Faculty of Medicine, University of Helsinki, Helsinki, Finland; 4 Sports Performance Research Institute New Zealand (SPRINZ), Auckland University of Technology, Auckland, New Zealand; 5 Department of Exercise Science and Health Promotion, Muscle Physiology Research Laboratory, Florida Atlantic University, Boca Raton, FL 33431 USA

**Keywords:** Energy availability, IGF-1, Weight loss, Bodybuilding, POMS, REDs

## Abstract

**Purpose:**

Physique athletes engage in rigorous competition preparation involving intense energy restriction and physical training to enhance muscle definition. This study investigates hormonal changes and their physiological and performance impacts during such preparation.

**Methods:**

Participants included female (10 competing (COMP) and 10 non-dieting controls (CTRL)) and male (13 COMP and 10 CTRL) physique athletes. COMP participants were tested 23 weeks before (PRE), one week before (MID), and 23 weeks after the competition (POST). Non-dieting CTRL participants were tested at similar intervals. Measurements included body composition (DXA), muscle cross-sectional area (ultrasound), energy availability (EA) derived by subtracting exercise energy expenditure (EEE) from energy intake (EI) and dividing by fat-free mass (FFM), muscle strength, and various serum hormone concentrations (ACTH, cortisol, estradiol, FSH, IGF-1, IGFBP-3, insulin, and free and total testosterone and SHBG).

**Results:**

During the diet, EA (*p* < 0.001), IGF-1 (*p* < 0.001), IGFBP-3 (*p* < 0.01), and absolute muscle strength (*p* < 0.01–0.001) decreased significantly in both sexes in COMP. Decreases in IGF-1 were also associated with higher loss in FFM. In males, testosterone (*p* < 0.01) and free testosterone (*p* < 0.05) decreased, while SHBG (*p* < 0.001) and cortisol (*p* < 0.05) increased. Insulin decreased significantly only in males (*p* < 0.001). Mood disturbances, particularly increased fatigue in males (*p* < 0.05), highlighted the psychological strain of competition preparation. All these changes were restored by increased EA during the post-competition recovery period.

**Conclusion:**

Significant reductions in IGF-1 and IGFBP-3 during competition preparation may serve as biomarkers for monitoring physiological stress. This study offers valuable insights into hormonal changes, muscle strength, and mood state during energy-restricted intense training.

## Introduction

Physique athletes undergo rigorous competition preparation with intense energy restriction and physical training to enhance muscle definition. This preparatory phase is followed by a recovery period of 3–4 months, during which body composition and endocrine changes are typically restored (Hulmi et al. 2017; Rossow et al. 2013). The off-season focuses on muscle growth through balanced nutrition and resistance training (Mursu et al. 2023). Hormone profiles in elite athletes differ from usual reference ranges, potentially reflecting baseline differences between athletes and the general population as well as the impact of athletic training on the endocrine systems (Healy et al. 2014).

We previously reported that physique athletes experience significant hormonal changes during the competition preparation. These inevitable changes, likely caused by the combined factors of weight loss, training and dieting stress, and the sustained energy deficit, include reduced leptin and T3 levels and increased ghrelin concentrations, reflecting shifts in energy metabolism (Isola et al. 2023). Weight loss also alters serum cardiometabolic profiles, such as insulin, highlighting metabolic complexities (Jouhki et al. 2024; Mäestu et al. 2010). Additionally, male athletes experience fluctuations in insulin-like growth factor 1 (IGF-1), free (FT), and total testosterone (T), while female athletes demonstrate changes in estradiol levels during the preparation (Hulmi et al. 2017; Mitchell et al. 2018).

Athletes may also experience reductions in muscular function, strength, and power as the focus shifts toward achieving ideal body aesthetics sometimes at the expense of exercise performance (Robinson et al. 2015). Additionally, bone mineral density (BMD) can decrease during periods of energy deficit, but it often recovers during refeeding (Hulmi et al. 2017). The physiological stress from the competition preparation may lead to disruptions in sleep patterns, mood disturbances, and alterations in nutrient intake, which collectively contribute to diminished exercise performance (Longstrom et al. 2020).

The concept of relative energy deficiency in sport (RED-S) expands our understanding of low energy availability (LEA) impacts. Affecting both male and female athletes, RED-S leads to hormonal imbalances, decreased bone mineral density, and weakened immune function. Energy deficiency also reduces muscle strength, power, and endurance, and can cause mood disturbances, anxiety, and depression. These changes impair reproductive function, metabolism, and overall health, causing conditions like hypothalamic amenorrhea in females and reduced fertility in males. Ultimately, RED-S compromises health, well-being, and athletic performance (Angelidi et al. 2024).

This study examined serum hormonal changes in physique athletes during the competition preparation. Understanding hormonal alterations vital to training and health outcomes is paramount, as it may inform coaching strategies. This research delves into how energy restriction and intense training impact hormone profiles, physiological performance, and psychological well-being, using the profile of mood states (POMS) to assess mood changes. We hypothesized that the competition diets would lead to changes in serum hormones, BMD, performance, and mood in both sexes. These findings may potentially improve coaching methods for supporting athletes while minimizing adverse effects from competition preparation.

## Materials and methods

### Participants

As previously detailed by Isola et al. (2023), 89 amateur physique athletes were recruited through various online platforms. Adhering to inclusion criteria, participants were amateur competitors (COMP) aiming for fat loss while maintaining muscle mass for the national championships or were non-competing controls (CTRL). The exclusion criteria ruled out individuals with chronic diseases and medication affecting the measured variables, junior or master competitors, and those not adhering to WADA guidelines. CTRL training background mirrored that of COMP. The study was ethically approved by the Ethics Committee of the Central Finland Health Care District (19U/2018), Finland and registered (ClinicalTrials.gov ID: NCT04392752), spanned from 2019 to 2020, and adhered to the Declaration of Helsinki.

### Study design

This study spanned 46 weeks, during which participants managed their own diet and exercise regimens. They attended three laboratory sessions at specific stages: 23 weeks before the competitions (PRE), one week before the competitions (MID) after an average of 21 weeks of dieting, and 23 weeks after the competitions (POST). Testing procedures were standardized to control for time of day, an overnight fast, and abstention from physical activities before assessments. Participants were provided a standardized meal after initial tests to mitigate the influence of dietary variables. For those traveling considerable distances, overnight accommodations were provided to ensure adherence to pre-test protocols, safeguarding data integrity.

### Resistance and aerobic training

Participants documented alterations in their training diaries (see Isola et al. 2023). Resistance training was quantified in the total weekly sets for each muscle group, and aerobic training as total minutes per week. The complete training data was available for a subset of COMP across different study phases, providing insights into the training volumes undertaken during the competition and recovery periods.

### Energy availability

Building upon the procedures delineated by Isola et al. (2023), this study further scrutinized the energy dynamics among physique athletes through EI tracking from nutrition logs and calculated EEE from detailed training logs. Participants’ weekly average training load was quantified using metabolic equivalents (MET), adhering to Ainsworth et al. (2000) guidelines, and converted into daily average EEE. These figures were juxtaposed with fat-free mass (FFM) changes from dual-energy X-ray absorptiometry (DXA), to provide a comprehensive view of energy availability (EA) during the competition preparation.

### Muscular performance assessment

The strength measurements were conducted 2–4 h after participants arrived at the study site. Participants received detailed procedural instructions and performed a standardized warm-up of 20 bodyweight squats and 10 lunges per leg. Isometric and concentric maximal voluntary forces were assessed using a strain gauge sensor on an isokinetic knee extension device.

The isokinetic knee extension dynamometer, custom-built by the Faculty of Sport and Health Sciences at the University of Jyväskylä, Finland, was adjusted to fit each participant. The device’s axis was aligned with the lateral condyle of the right knee, and the shin support was positioned above the ankle. Participants were secured with four-point belts to prevent movement, and all equipment adjustments were documented for consistency in the subsequent assessments.

The force measurement protocol included six dynamic repetitions and one isometric repetition. The sensor moved through a 70° range of motion (approximately 100°–170°, varying with individual proportions) at the angular velocity of 60°/s, pausing for 2 s at both the top and bottom positions. The first repetition involved no force to familiarize participants with the motion. During repetitions 2–6, participants exerted maximum effort during the isometric (bottom) and concentric (upward) phases, with no force exerted at the top or during the downward motion. After these dynamic repetitions, participants performed one additional maximal isometric contraction for about 2 s. Verbal instructions such as "press" for maximal effort and "stop" to cease exertion were provided throughout the measurements.

Before the assessment, participants completed a familiarization session whereby they exerted no torque during the first repetition, then ~ 20% of estimated maximum voluntary torque during repetitions 2–6 and ~ 80% during repetition 7. Isokinetic knee extension data were recorded using the Spike 2™ software (Cambridge Electronic Design, Cambridge, UK), capturing torque and angle changes. Post-measurement, raw data were processed using an IIR filter (Low pass Chebyshev 2) to remove interfering signals. The analysis focused on the maximum force produced during isometric contractions and the concentric phase, with peak torque not necessarily occurring during the first repetition. The best results for isometric maximum force and concentric torque were analyzed, along with a fatigue profile based on the performance from the first actual repetition to the last repetition. The fatigue profile was analyzed for both isometric force and concentric torque.

### Bone mass and mineral density

Body composition and bone Z-score were estimated by dual-energy X-ray absorptiometry (DXA) (Lunar Prodigy Advance EnCore version 14.10.022, GE Medical Systems—Lunar, Madison WI USA), as described previously (Isola et al. 2023). The Z-score represents the standard deviations by which the study participants’ BMD deviates from the average BMD of a control group matched for age and sex (Carey et al. 2007).

### Ultrasound for muscle cross-sectional area

A muscle cross-sectional area (CSA) of vastus lateralis (VL) was examined at the mid-thigh using a B-mode axial plane ultrasound (model SSD-α10, Aloka, Tokyo, Japan) with a 10 MHz linear-array probe (60 mm width) in the extended-field-of-view mode (23 Hz sampling frequency). The accuracy and validity of the measurements have been confirmed in a prior study (Isola et al. 2023).

### Blood parameters

Venous blood samples were obtained from the antecubital vein and put into serum tubes (Venosafe; Terumo Medical Co., Leuven, Hanau, Belgium). Samples were stored at room temperature for 30 min before being centrifuged at 3500 rpm for 10 min (Megadure 1.0 R Heraeus; DJB Lab Care, Hanau, Germany). IGF-1, Insulin-like Growth Factor Binding Protein 3 (IGFBP-3), Adrenocorticotropic Hormone (ACTH), Follicle-Stimulating Hormone (FSH), Sex Hormone-Binding Globulin (SHBG), T, Free Testosterone (FT), Cortisol (C), Insulin, and Estradiol were analyzed from serum using the Siemens Immulite 2000 XPi immunoassay system (Siemens Healthineers, Erlangen, Germany). Estradiol was analyzed using Immulite® 2000 (L2KE2-17) commercial kits. FT was tested by ELISA using the DS2 automated ELISA system from Dynex Technologies (Testosterone Free ELISA, REF DE2924). The lowest detectable level of insulin was 0.042 pmol/l. Participants with insulin levels below this threshold (9 out of 45) were recorded as having 0.042 pmol/l. The sensitivity of the ACTH assay was 1.1 pmol/L, with an intra-assay coefficient of variation (CV) of 8.7% and an inter-assay CV of 10%. The testosterone assay had a sensitivity of 0.5 nmol/L, with intra-assay and inter-assay CVs of 16.3% and 24.3%, respectively. Insulin had a sensitivity of 0.042 mIU/L, with intra-assay and inter-assay CVs of 5.5% and 7.3%, respectively. The cortisol assay had a sensitivity of 5.5 nmol/L, with an intra-assay CV of 6.1% and an inter-assay CV of 8.2%. IGF-1 was detected with a sensitivity of 2.6 nmol/L, displaying intra-assay and inter-assay CVs of 3.9% and 7.7%, respectively. IGFBP-3 had a sensitivity of 100 ng/mL, with intra-assay and inter-assay CVs of 4.4% and 6.6%, respectively. Estradiol sensitivity was 55 pmol/L, with an intra-assay CV of 9.9% and an inter-assay CV of 16%. The FSH assay exhibited a sensitivity of 0.1 IU/L, with intra-assay and inter-assay CVs of 2.9% and 4.1%, respectively. The SHBG assay sensitivity was 0.2 nmol/L, with intra-assay and inter-assay CVs of 2.5% and 4.2%. Finally, the free testosterone (FT) assay had a sensitivity of 0.018 pg/mL, with intra-assay and inter-assay CVs of 7% and 12%, respectively. Participants with insulin levels below the threshold (0.042 mIU/L) were assigned a value of 0.042 mIU/L (nine out of 45 participants). The testosterone–cortisol ratio (T/C ratio) was calculated as [T (nmol l − 1)/C (nmol l − 1)]. We excluded female participants using any form of contraceptive from the final analyses of ACTH, FSH, estradiol, and SHBG.

### POMS and reproductive function questionnaires

The mood and menstrual function of female participants were evaluated monthly throughout the study, focusing on aligning these assessments with laboratory visits at the PRE, MID, and POST time points for analysis. Mood states were evaluated using the Finnish adaptation (Vuoskoski and Eerola 2011) of the POMS-Adolescents (POMS-A; Terry et al. 1999). The POMS-A comprises 24 items that appraise six distinct affective dimensions: vigor, confusion, anger, fatigue, depression, and tension. Responses were provided on a 5-point Likert scale, where 1 signifies ‘not at all’ and 5 denotes ‘extremely.’ The investigation into the menstrual status and the use of hormonal contraception, including oral contraceptives and intrauterine devices, was conducted through questionnaires. The participants completed and submitted these questionnaires at the time of measurement.

### Statistical analysis

We used IBM SPSS for statistical analysis and tested data normality with the Shapiro–Wilk test. COVID-19 impacted our ability to complete POST measurements for all participants, leading to an initial ANOVA or Mann–Whitney test to compare groups at PRE. We then assessed changes from PRE to MID and MID to POST within groups using paired t-tests or Wilcoxon tests, depending on data distribution. Subsequently, ANOVA assessed absolute differences between COMP and CTRL across these periods. Differences between female and male COMP from PRE to MID and to POST and percentage changes in hormones, *Z*-score, and torque variables between sexes were also examined with ANOVA. Pearson’s or Spearman’s coefficients analyzed correlations between variables of interest, setting statistical significance at *p* < 0.05. This approach builds on the methodologies from prior research (Isola et al. 2023).

## Results

### Body composition, energy intake and bone health

Our earlier findings showed that both sexes experienced similar reductions in EI, body mass (BM) and fat mass (FM) as well as a slight decrease in muscle size throughout the 21-week competition preparation (Isola et al. 2023). However, a distinct outcome was noted among males, who uniquely exhibited a loss in lean mass (LM). Instead of LM, the present study employed FFM to calculate EA. Given that FFM’s behavior mirrored LM, we have not reported these measures separately.

**Fig. 1:**
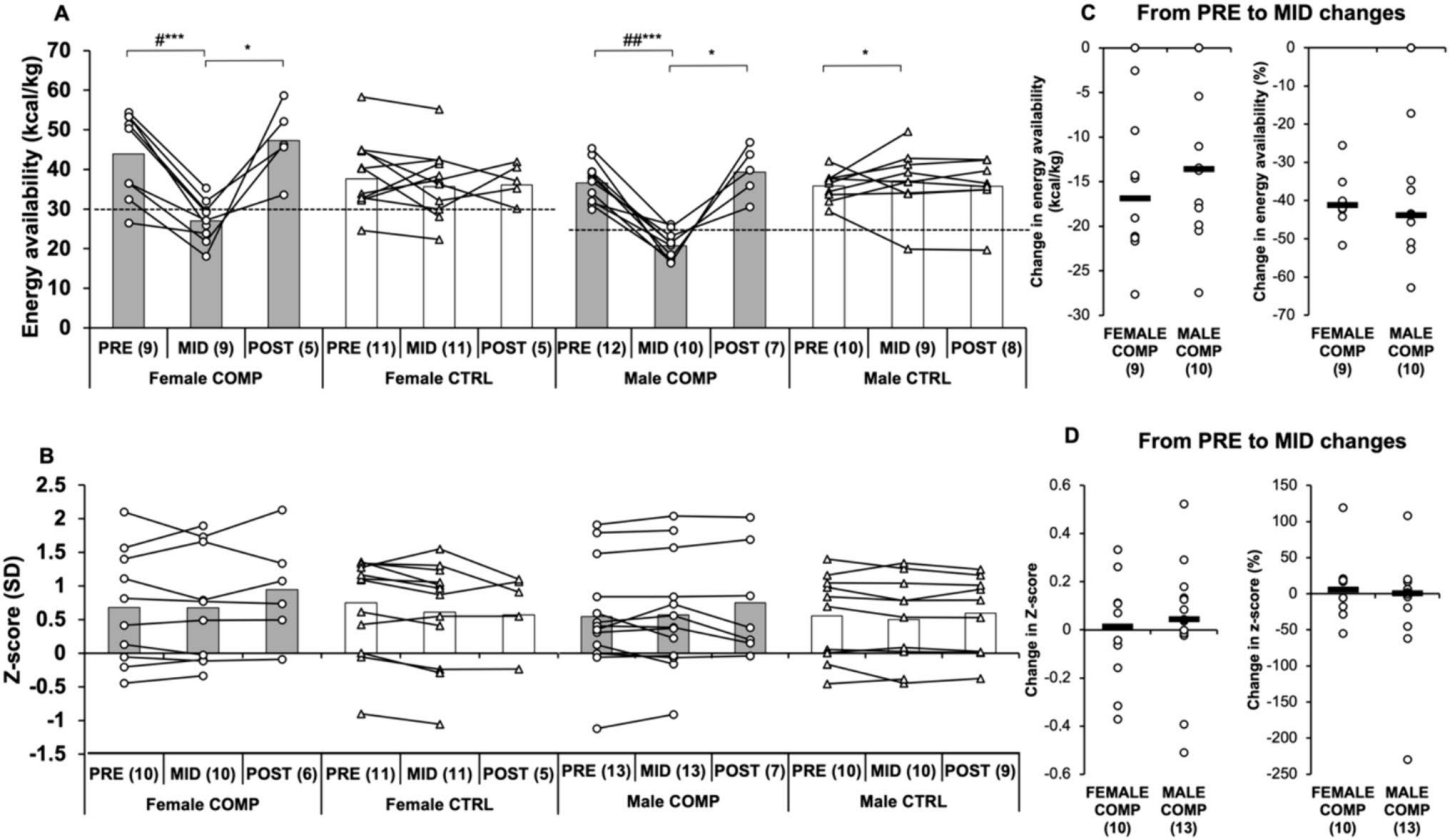
**A**, **B** Absolute changes in energy availability and bone mineral density in COMP and CTRL groups from PRE to MID and MID to POST. Baseline pre-test (PRE) was obtained before the dieting phase for the competition, MID one week before the competition, and POST after the recovery period. Numbers in parentheses indicate the number of participants. Circles and triangles indicate individual data for the COMP and CTRL groups, respectively, and bars indicate means. ∗ indicates a statistically significant change within the group. # indicates a statistically significant difference between the COMP and CTRL groups in the change. ∗ #, ∗ ∗ ##, and ∗∗∗ ### represent *p*-values ranging from < 0.05 to < 0.001. The dotted line represents the EA threshold. EA levels below 30 kcal/kg FFM per day are associated with significant disruptions in body systems, including hormonal alterations and bone health issues. **C**, **D** Absolute and percentage changes in female and male COMP groups from PRE to MID. Circles indicate individual data and black lines indicate the mean difference between sexes. Energy availability is expressed in kcal/kg, derived by subtracting EEE from EI and dividing by FFM. Z-score is used as an indicator of bone density

EA (derived by subtracting EEE from EI and dividing by FFM) decreased (*p* < 0.001) during the weight-loss period in both male and female COMP (from 43.9 ± 10.8 to 27.0 ± 5.28 kcal/kg and from 38.2 ± 6.9 kcal/kg to 20.8 ± 3.50 kcal/kg, respectively) and the change was significant compared to CTRL (*p* < 0.05, Fig. 1). EA increased in male CTRL (*p* < 0.05). Notably, EA in male COMP at MID was significantly lower than in female COMP (*p* < 0.01). EA increased in both COMP groups from MID to POST (*p* < 0.05) and recovered back to baseline.

*Z*-scores showed no significant changes from PRE to MID in either COMP group. Scores remained stable for female COMP participants (from 0.68 ± 0.85 to 0.68 ± 0.84) and for male COMP participants (from 0.75 ± 1.13 to 0.78 ± 1.02) (Fig. 1). Similarly, no significant changes were observed in any of the groups from MID to POST.

### Hormone profiles

**Fig. 2:**
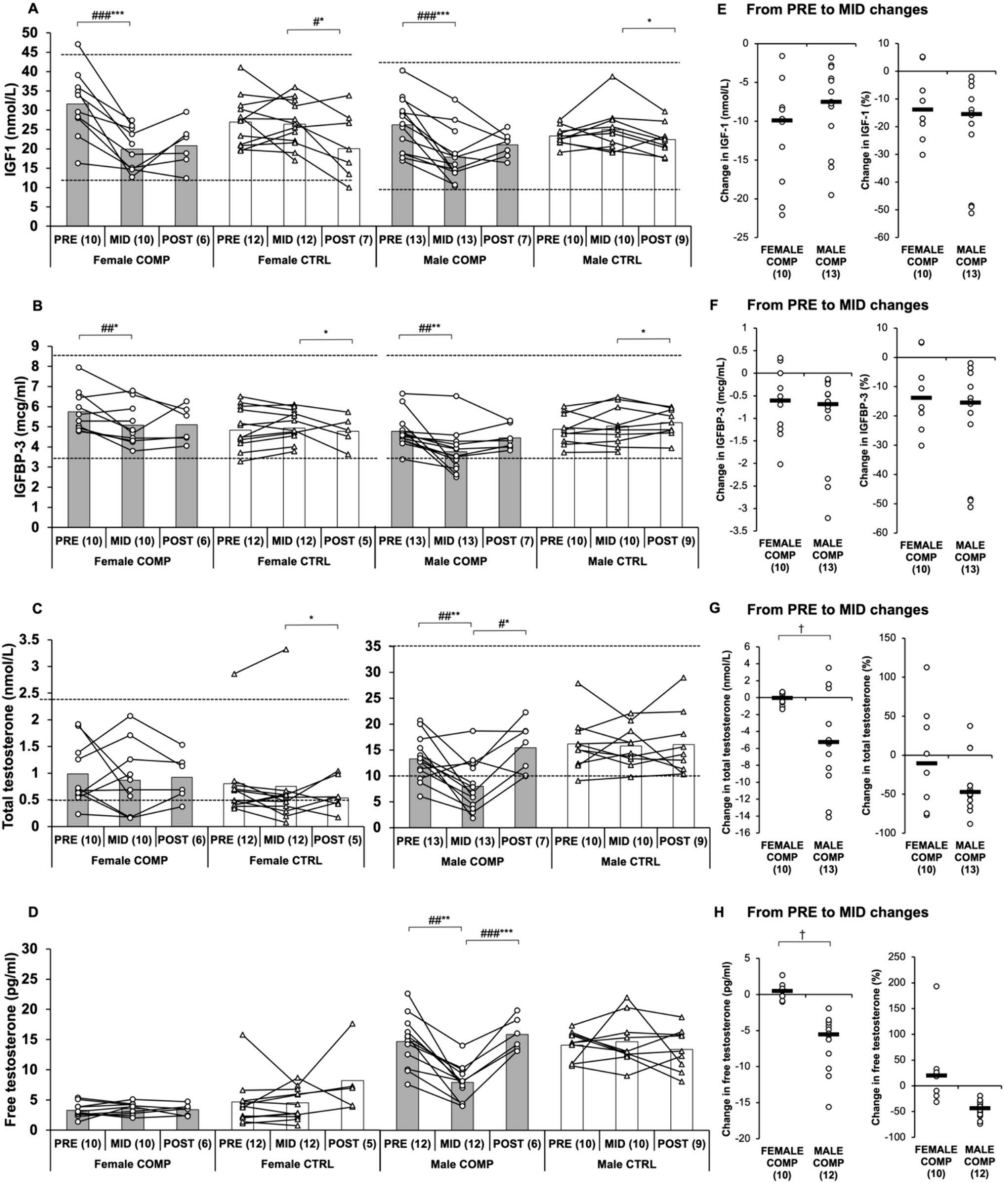
**A–D** Absolute changes in IGF-1, IGFBP-3, total testosterone, and free testosterone in COMP and CTRL groups from PRE to MID and MID to POST. Baseline pre-test (PRE) was obtained before the dieting phase for the competition, MID one week before the competition, and POST after the recovery period. Numbers in parentheses indicate the number of participants. Circles and triangles indicate individual data for the COMP and CTRL groups, respectively, and bars indicate means. ∗ indicates a statistically significant change within the group. # indicates a statistically significant difference between the COMP and CTRL groups in the change. ∗ #, *∗ ##, and ∗∗ ∗ ### represent *p*-values ranging from < 0.05 to < 0.001. The black dotted lines represent the reference values for a normal-weight person. **E–H** Absolute and percentage changes in female and male COMP groups from PRE to MID. Circles indicate individual data and black lines indicate the mean. † indicates a statistically significant (*p* < 0.05) difference between the sexes. IGF-1, insulin-like growth factor 1; IGFBP-3, insulin-like growth factor binding protein 3. Note: No reference values are provided for free testosterone because directly measured free testosterone levels can differ significantly from calculated values

**Fig. 3:**
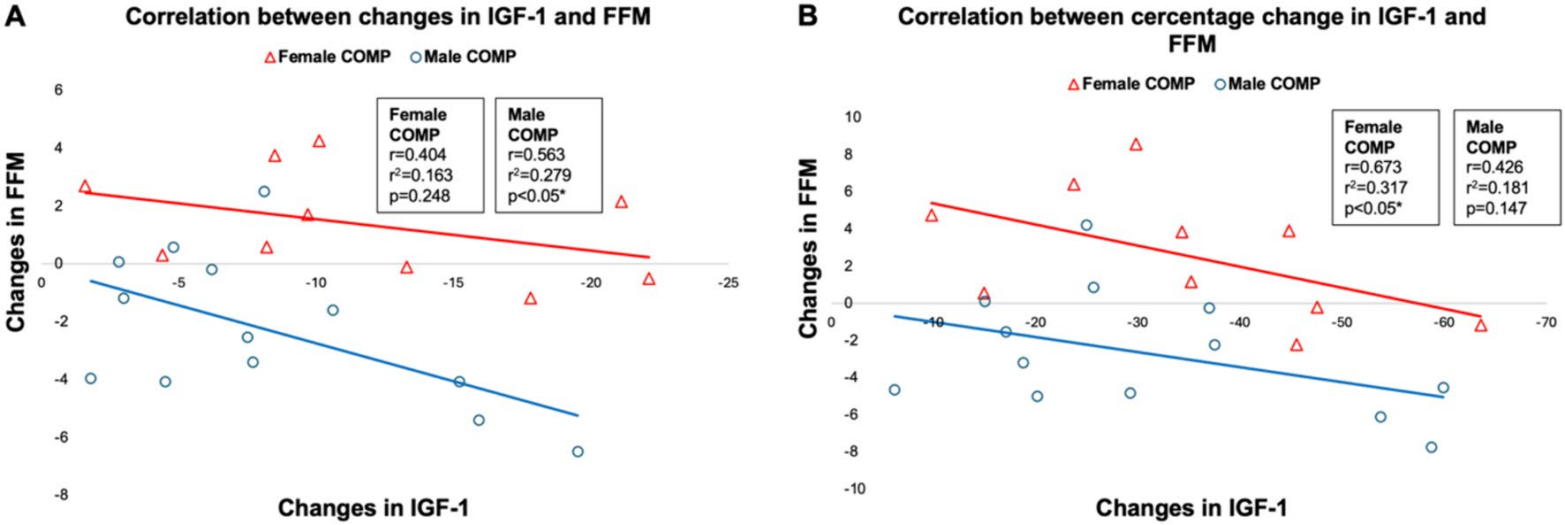
These scatter plots illustrate the relationship between changes in IGF-1 and changes in FFM in female (**A**, **C**) and male (**B**, **D**) COMP from PRE to MID. Each point represents an individual participant. Scatter plots **A** and **B** depict absolute changes, while scatter plots **C** and **D **depict changes in percentages

IGF-1 decreased significantly in both female and male COMP from PRE to MID compared to CTRL (*p* < 0.001, Fig. 2). There was a significant difference between sexes in COMP in IGFBP-3 levels at baseline (*p* < 0.05). Additionally, IGFBP-3 significantly decreased in female and male COMP groups (*p* < 0.01). A significant correlation was found between individual changes in IGF-1 and FFM in male COMP (*p* < 0.05, Fig. 3). The percentage change in IGF-1 and FFM measured by DXA also correlated significantly (*p* < 0.05). IGF-1 levels in both COMP groups did not significantly change from MID to POST. However, comparing female COMP to CTRL, the difference in IGF-1 levels was statistically significant (*p* < 0.05).

Testosterone (T) and free testosterone (FT) levels significantly differed between male COMP and female COMP at baseline (*p* < 0.001, respectively, Fig. 2). T and FT levels decreased in male COMP from PRE to MID (*p* < 0.01) and compared to CTRL (*p* < 0.05 and *p* < 0.001). There was a difference in T (*p* < 0.05) and FT (*p* < 0.01) levels between the sexes in COMP from PRE to MID. Both T (*p* < 0.001) and FT (*p* < 0.05) levels increased from MID to POST, recovering back to baseline, and these changes were also significant when compared to CTRL (*p* < 0.05 and *p* < 0.001, respectively). No significant changes were observed in the female groups across the study, except T decreased from MID to POST in female CTRL (*p* < 0.05).

**Fig. 4:**
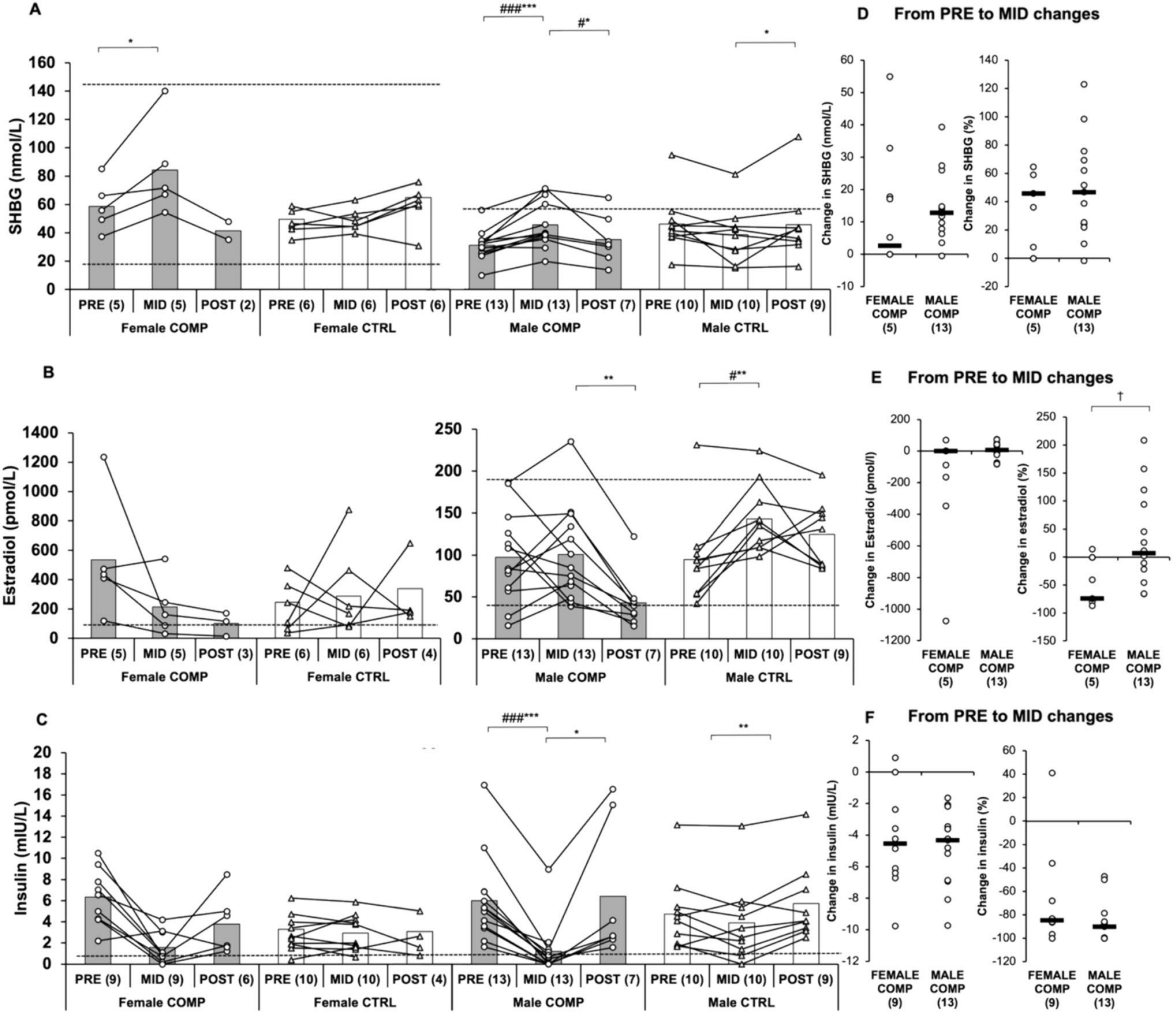
**A–C** Absolute changes in SHBG, estradiol, and insulin in COMP and CTRL groups from PRE to MID and MID to POST. Baseline pre-test (PRE) was obtained before the dieting phase for the competition, MID one week before the competition, and POST after the recovery period. Numbers in parentheses indicate the number of participants. Circles and triangles indicate individual data for the COMP and CTRL groups, respectively, and bars indicate means. ∗ indicates a statistically significant change within the group. # indicates a statistically significant difference between the COMP and CTRL groups in the change. ∗ #, ∗∗ ##, and ∗∗∗ ### represent *p*-values ranging from < 0.05 to < 0.001. The black dotted lines represent the reference values or at least the lower reference value for a normal-weight person. **D–F** Absolute and percentage changes in female and male COMP groups from PRE to MID. Circles indicate individual data and black lines indicate the mean. † indicates a statistically significant (*p* < 0.05) difference between the sexes. *SHBG* sex hormone-binding globulin

SHBG differed between sexes at baseline in COMP (*p* < 0.001). SHBG increased in both female (*p* < 0.05) and male (*p* < 0.001) COMP from PRE to MID (Fig. 4), but only male COMP differed significantly from CTRL (*p* < 0.001). From MID to POST, SHBG levels decreased in male COMP (*p* < 0.05) and increased in male CTRL (*p* < 0.05), with the change being significant (*p* < 0.05). No significant changes were observed in female groups.

No significant changes were observed in estradiol levels in female and male COMP. There was a statistically significant difference in percentage change between the sexes in COMP from PRE to MID (*p* < 0.05). No significant changes were observed in either female or male COMP from PRE to MID, but in male CTRL, estradiol increased (*p* < 0.01) significantly compared to COMP (*p* < 0.05). From MID to POST, estradiol significantly decreased in male COMP (*p* < 0.01, Fig. 4). In contrast, no changes were observed in the female groups.

Insulin decreased in male COMP (*p* < 0.001), and the change was statistically significant (*p* < 0.001, Fig. 4) compared to CTRL from PRE to MID. There was a significant difference in insulin between female COMP and CTRL at baseline (*p* < 0.01). However, no significant change was observed in insulin from PRE to MID within female COMP or CTRL. In contrast, male COMP experienced a statistically significant reduction in insulin from PRE to MID (*p* < 0.001), which was significantly different compared to male CTRL (*p* < 0.001).

**Fig. 5:**
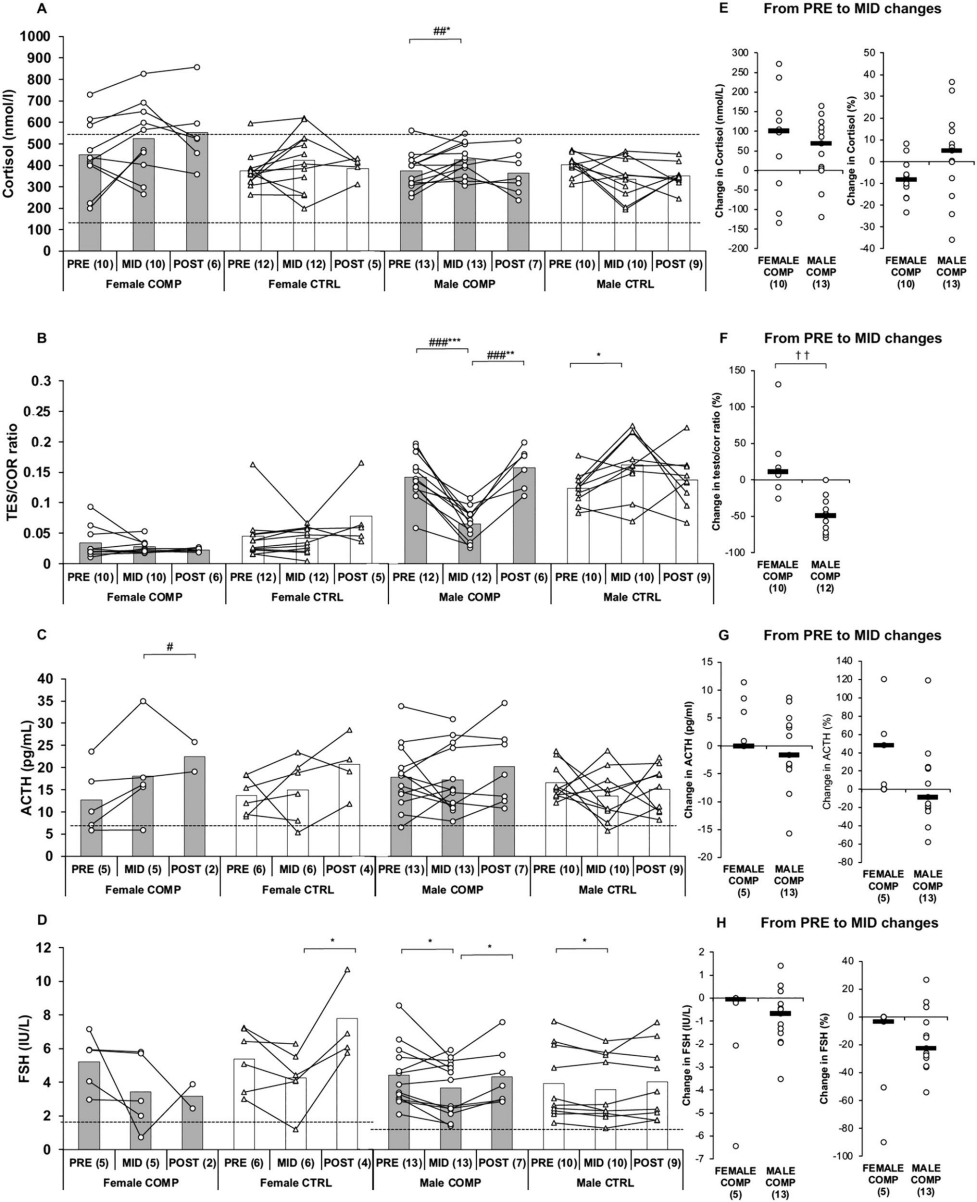
**A–D** Absolute changes in cortisol, TES/COR ratio, ACTH, and FSH in COMP and CTRL groups from PRE to MID and MID to POST. Baseline pre-test (PRE) was obtained before the dieting phase for the competition, MID one week before the competition, and POST after the recovery period. Numbers in parentheses indicate the number of participants. Circles and triangles indicate individual data for the COMP and CTRL groups, respectively, and bars indicate means. ∗ indicates a statistically significant change within the group. # indicates a statistically significant difference between the COMP and CTRL groups in the change. ∗ #, ∗∗ ##, and ∗∗∗ ### represent *p*-values ranging from < 0.05 to < 0.001. The black dotted lines represent the reference values or at least the lower reference value for a normal-weight person. **D–F** Absolute and percentage changes in female and male COMP groups from PRE to MID. Circles indicate individual data and black lines indicate the mean. †† indicates a statistically significant (*p* < 0.01) difference between the sexes. *TES/COR ratio*, testosterone and cortisol ratio, *ACTH* adrenocorticotropic hormone, *FSH* follicle-stimulating hormone

Cortisol increased significantly in male COMP from MID to POST (*p* < 0.05, Fig. 5), but there was no significant difference compared to CTRL. No significant changes in cortisol were observed in the female groups.

The T/C ratio significantly decreased in male COMP (*p* < 0.001, Fig. 5), and the change was significant compared to CTRL (*p* < 0.001). No significant changes were observed in the female groups. Male COMP experienced a decrease of -50.4 ± 17.8%, which was more pronounced (*p* < 0.01) compared to female COMP (6.8 ± 53.5%). From MID to POST, T/C ratio increased in male COMP (*p* < 0.01), and the change was significant compared to CTRL (*p* < 0.001). Additionally, there was a significant difference between sexes (*p* < 0.001) in COMP.

Follicle-stimulating hormone decreased in male COMP and CTRL (*p* < 0.05) from PRE to MID. FSH increased in male COMP (*p* < 0.05) and female CTRL (*p* < 0.05) from MID to POST (Fig. 5). No significant changes were observed in ACTH, except in female COMP from MID to POST.

### Strength measurements

**Fig. 6:**
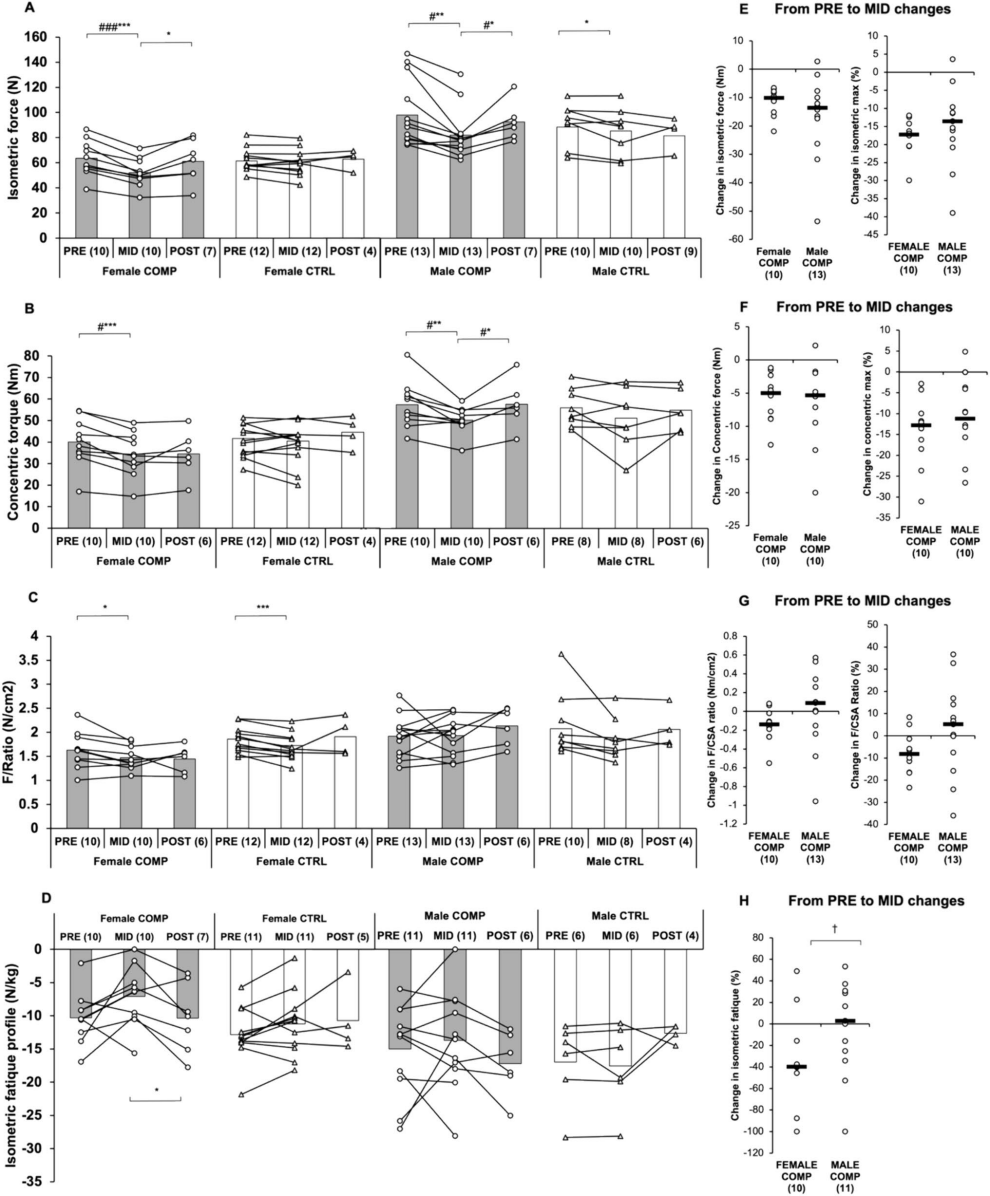
**A–D** Absolute changes in lower body strength in COMP and CTRL groups from PRE to MID and MID to POST. Baseline pre-test (PRE) was obtained before the dieting phase for the competition, MID one week before the competition, and POST after the recovery period. Numbers in parentheses indicate the number of participants. Circles and triangles indicate individual data for the COMP and CTRL groups, respectively, and bars indicate means. ∗ indicates a statistically significant change within the group. # indicates a statistically significant difference between the COMP and CTRL groups in the change. ∗ #, ∗∗ ##, and ∗ ∗∗ ### represent *p*-values ranging from < 0.05 to < 0.001. The black dotted lines represent the reference values or at least the lower reference value for a normal-weight person. **D–F** Absolute and percentage changes in female and male COMP groups from PRE to MID. Circles indicate individual data and black lines indicate the mean. † indicates a statistically significant (*p* < 0.05) difference between the sexes. F/Ratio, the ratio of maximal isometric muscle force to CSA (F/CSA); Isometric fatigue profile, fatigue profile based on the performance from the first actual repetition to the last repetition

Isometric force decreased from PRE to MID in both female (*p* < 0.001) and male (*p* < 0.01) COMP and male CTRL (*p* < 0.05, Fig. 6). Additionally, isometric force was lower in COMP compared to CTRL in both sexes (*p* < 0.001 and *p* < 0.05, respectively). Concentric torque also decreased from PRE to MID in both female and male COMP (*p* < 0.001 and *p* < 0.01, respectively) and was lower compared to CTRL (*p* < 0.05). The ratio of maximal isometric muscle force to CSA (F/CSA) decreased in female COMP (*p* < 0.05) and female CTRL (*p* < 0.001). No significant changes were observed in the male groups. A significant difference was noted in the changes in isometric fatigue profile between sexes from PRE to MID (*p* < 0.05). Isometric fatigue decreased in female COMP from MID to POST (*p* < 0.05). No significant changes were observed in the concentric fatigue profiles in any of the groups (data not shown).

### Menstrual irregularities

In female COMP, 50% of participants (5/10) reported using hormonal contraception, with one experiencing a disturbed menstrual cycle between the PRE and MID phases. Additionally, three members of COMP who did not use hormonal contraception reported irregular menstruation during the same period. Thus, four participants in COMP experienced menstrual irregularities during the study period. In female CTRL, 50% of participants (6/12) were using hormonal contraception, and four experienced irregular menstrual irregularities throughout the study period.

### POMS

Male COMP experienced an increase in FATIGUE (*p* < 0.05), and the change was significant compared to CTRL (*p* < 0.01, Table 1). TENSION also increased (*p* < 0.05), but this change was not significantly different compared to CTRL. However, significant sex differences were noted in TENSION changes from PRE to MID (*p* < 0.01), where female TENSION decreased. No significant difference was noted in female COMP groups. From MID to POST, FATIGUE decreased significantly (*p* < 0.01), and DEPRESSION decreased (*p* < 0.05) in male COMP, but these changes were not significantly different from CTRL. No significant changes were observed in female groups.

**Table 1:** Profile of mood states

POMS	*n*	Pre	Mid	Change	*n*	Mid	Post	Change
Vigor								
Female COMP	9	3.19 ± 0.89	3.08 ± 1.16	−0.11 ± −0.26	5	2.70 ± 1.02	2.75 ± 0.73	0.05 ± 0.29
Male COMP	12	3.13 ± 0.76	2.77 ± 0.82	−0.35 ± −0.06	8	3.06 ± 0.73	3.31 ± 1.08	0.25 ± 0.35
Female CTRL	12	3.41 ± 0.64	3.16 ± 0.97	−0.25 ± −0.33	8	3.06 ± 0.87	3.16 ± 1.03	0.09 ± 0.16
Male CTRL	10	3.20 ± 0.90	3.18 ± 1.01	−0.03 ± −0.11	9	3.00 ± 0.90	2.97 ± 0.32	−0.03 ± 1.04
Confusion								
Female COMP	9	1.36 ± 0.42	1.31 ± 0.66	−0.06 ± −0.24	5	1.05 ± 0.11	1.20 ± 0.27	0.15 ± 0.16
Male COMP	12	1.38 ± 0.42	1.44 ± 0.63	0.05 ± −0.14	8	1.34 ± 0.72	1.38 ± 0.63	0.03 ± 0.09
Female CTRL	12	1.41 ± 0.42	1.43 ± 0.57	0.02 ± −0.15	8	1.47 ± 0.60	1.59 ± 1.13	0.13 ± 0.52
Male CTRL	10	1.28 ± 0.36	1.30 ± 0.52	0.03 ± −0.16	9	1.33 ± 0.54	1.31 ± 0.45	−0.03 ± 0.10
Depression								
Female COMP	9	1.11 ± 0.25	1.08 ± 0.18	−0.03 ± 0.08	5	1.05 ± 0.11	1.20 ± 0.33	0.15 ± 0.38 †
Male COMP	12	1.27 ± 0.62	1.42 ± 0.58	0.15 ± 0.05	8	1.28 ± 0.39	1.13 ± 0.27	−0.16 ± 0.12
Female CTRL	12	1.18 ± 0.25	1.20 ± 0.33	0.02 ± −0.08	8	1.22 ± 0.36	1.31 ± 0.48	0.06 ± 0.12
Male CTRL	10	1.43 ± 0.50	1.45 ± 0.61	0.02 ± −0.11	9	1.50 ± 0.63	1.17 ± 0.33	−0.33 ± 0.29*
Anger								
Female COMP	9	1.11 ± 0.18	1.19 ± 0.30	0.08 ± 0.12	5	1.15 ± 0.68	1.05 ± 0.11	−0.10 ± 0.22
Male COMP	12	1.52 ± 0.60	1.63 ± 0.64	0.10 ± −0.05	8	1.56 ± 0.72	1.31 ± 0.59	−0.25 ± 0.12
Female CTRL	12	1.11 ± 0.17	1.32 ± 0.40	0.20 ± −0.23*	8	1.41 ± 0.44	1.22 ± 0.28	−0.19 ± 0.16
Male CTRL	10	1.30 ± 0.45	1.48 ± 0.66	0.18 ± −0.21	9	1.53 ± 0.68	1.19 ± 0.39	0.33 ± 0.29
Tension								
Female COMP	9	1.42 ± 0.41	1.11 ± 0.25	−0.31 ± 0.16 ††	5	1.05 ± 0.11	1.35 ± 0.42	0.30 ± 0.31
Male COMP	12	1.40 ± 0.51	1.60 ± 0.69	0.21 ± −0.18	8	1.53 ± 0.67	1.34 ± 0.57	−0.19 ± 0.11
Female CTRL	12	1.43 ± 0.50	1.45 ± 0.37	0.02 ± 0.13	8	1.46 ± 0.39	1.60 ± 0.73	0.15 ± 0.34
Male CTRL	10	1.18 ± 0.26	1.40 ± 0.57	0.23 ± −0.30	9	1.44 ± 0.58	1.31 ± 0.48	−0.14 ± 0.10
Fatigue								
Female COMP	9	1.58 ± 0.52	1.69 ± 0.83	0.11 ± −0.31	5	1.55 ± 0.21	1.60 ± 0.65	0.05 ± 0.60
Male COMP	12	1.75 ± 0.53	2.21 ± 0.42	+0.46 ± 0.11*##	8	2.06 ± 0.40	1.56 ± 0.55	−0.50 ± 0.15*
Female CTRL	12	1.75 ± 0.49	1.80 ± 0.46	0.05 ± 0.03	8	1.78 ± 0.39	1.66 ± 0.46	−0.13 ± 0.07
Male CTRL	10	2.08 ± 0.91	1.80 ± 0.90	−0.28 ± 0.02	9	1.89 ± 0.90	1.56 ± 0.43	−0.33 ± 0.47

Mean ± SD for all values. * indicates a statistically significant change within the group. # indicates a statistically significant difference between the COMP and CTRL groups in the change. ∗ , ∗∗ , ∗∗ ∗ represent *p* values < 0.05, < 0.01, and < 0.001, respectively. #, ##, ### represent *p* values < 0.05, < 0.01, and < 0.001, respectively. † indicates a statistically significant (*p* < 0.05) difference between the sexes. †† indicates a statistically significant (*p* < 0.01) difference between the sexes

## Discussion

We hypothesized that competition diets would lead to changes in serum hormones, BMD, performance, and mood in both sexes. As expected, significant changes in serum hormone levels were observed, indicating energy deficiency, and these changes were in part sex-specific. While mood and strength changes were observed, no changes in BMD were detected. A primary finding was the reduction in IGF-1 and its binding protein IGFBP-3 among both sexes during the competition preparation, suggesting that these may serve as potential biomarkers for monitoring physiological stress (Angelidi et al. 2024).

Insulin-like growth factor 1, a crucial hormone for muscle development (Yoshida and Delafontaine 2020), significantly decreased in the female and male COMP groups. Similarly, IGFBP-3, which modulates IGF-1 bioavailability, also decreased. This observation aligns with Mitchell et al. (2018), who reported similar reductions in IGF-1 among male physique athletes during competition preparation. Nutrition plays a crucial role in regulating circulating IGF-1 levels, with energy restriction profoundly impacting serum concentrations (Henning et al. 2014). In this study, the reduction in IGF-1 and IGFBP-3 may be attributed to reduced EI and LEA during the competition preparation. We previously reported an association between IGF-1 levels and EA at baseline before the competition preparation among a larger sample of physique athletes (Mursu et al. 2023).

We observed correlations between IGF-1 levels and changes in FFM in male and female COMP. This supports the argument that IGF-1 may be a potential biomarker to monitor physiological stress during the intense training period and nutritional changes that alter body composition, such as physique competition preparation. It also aligns with previous studies in soldiers; Nindl et al. (2007) demonstrated that IGF-1 and IGFBP-3 align with energy deficits and body composition changes, with free IGF-1 positively correlating with changes in body mass and FFM. Additionally, IGF-1 has a role in facilitating protein synthesis and muscle growth, and low levels of IGF-1 are associated with reduced BMD (Yan et al. 2016).

There was LEA during competition preparation in both female and male COMP groups with no significant sex differences. Despite LEA, no significant changes were observed in BMD, aligning with findings by Schoenfeld et al. (2023) in their systematic review of competitors during preparation for physique competitions. However, a study on elite endurance athletes revealed that LEA can reduce BMD (Heikura et al. 2018). Furthermore, Ihle and Loucks (2004) conducted a 5-day intervention manipulating EI in young females, resulting in significant effects on bone formation and resorption. Our results suggest that a 21-week weight loss period may not, on average, negatively impact bone health in physique athletes despite low EI and reduced IGF-1 levels. One explanation for this may be that physique athletes engage in extensive strength training, which is known to protect bone mineral content and density by stimulating new bone formation in areas subjected to mechanical strain (Pal et al. 2023).

We observed a significant reduction in insulin in male COMP, with a similar but not statistically significant trend in females. This aligns with Mäestu et al. (2010), who documented significant insulin declines in male bodybuilders during competition preparation. On the other hand, this was not observed in the study by Mitchell et al. (2018) who found no significant changes in insulin levels. The decrease in insulin was accompanied by reduced blood glucose as reported by us earlier from this cohort. (Jouhki et al. 2024).

Sex hormone changes among physique athletes appear to be driven by sex-specific physiological responses to energy deficits. In male COMP, T and FT decreased, consistent with hormonal adaptation patterns observed by Mäestu et al. (2010). These decreases are likely due to hypothalamic–pituitary–gonadal (HPG) axis disruptions. At the same time, males often experience more pronounced disruptions in this axis under energy deficits than females, leading to greater reductions in T (Pasiakos et al. 2019). No significant changes in T and FT were observed in female COMP, highlighting the different physiological responses to competition preparation. This finding contrasts Hulmi et al. (2017) and Mero et al. (2010), who reported decreases in T among females under weight loss conditions. One explanation for the potential sex differences might be that males exhibit reductions in T and FT due to their higher initial levels (and possibly greater reliance on these hormones for muscle maintenance), while females might experience changes in menstrual cycle regularity and function due to changes in estrogen levels rather than T during the weight-loss period (Williams et al. 2015). T and FT levels returned to baseline in male COMP after recovery.

SHBG increased in male and female COMP during competition preparation. Significant weight loss achieved through reduced caloric intake has previously led to increased SHBG in both female (Mero et al. 2010) and male athletes (Karila et al. 2008). Elevated SHBG levels suggest that less FT is available to tissues because more T is bound to SHBG, with approximately 65% of T being bound to SHBG (Allen et al. 2002). When high SHBG concentrations are combined with lower T levels, it likely indicates a lower availability of FT. An earlier study conducted in our laboratory reported that during high-volume strength training periods, decreases occurred in serum T and in the T/SHBG ratio in male weightlifters, with individual changes in the T/SHBG ratio correlating with changes in weightlifting performance (Häkkinen et al. 1987). Additionally, baseline serum basal T concentrations in males (Ahtiainen et al. 2003) and the serum testosterone/SHBG ratio and FT in females have been shown to correlate with individual changes in maximal strength and/or muscle cross-sectional area both in the lower and upper body during the strength training period (Häkkinen et al. 1992, 2022). These findings highlight the importance of monitoring serum SHBG and T levels in physique athletes, as they may play a critical role in maintaining muscle mass and optimizing performance during training periods and competition preparation. Ensuring adequate energy availability, modifying training intensity and volume to reduce physiological stress during competition preparation, and incorporating strategies to enhance recovery could mitigate these hormonal disruptions.

Our analysis of cortisol in female athletes suggested an upward trend, although not statistically significant. This observation is nuanced, given mixed literature results. Hulmi et al. (2017) found basal cortisol was not significantly changed in female physique athletes, while Mitchell et al. (2018) also reported no significant change in C in male physique athletes. Elevation of cortisol is typically linked to LEA, suggesting an adaptive response to energy stress (Mountjoy et al. 2023).

In our study, the significant decrease in the T/C ratio in male COMP during competition preparation likely indicates increased physiological stress and an imbalance between anabolic and catabolic processes. The T/C ratio may be a valuable marker for assessing this balance and optimizing training loads and recovery strategies, especially during prolonged training periods lasting several weeks to months, such as competition preparation (De Luccia 2016; Häkkinen et al. 1985). However, the interaction between the hypothalamic–pituitary–adrenal (HPA) axis and the HPG axis may vary between sexes, influencing hormonal responses and adaptations during periods of energy deficit and stress (Pasiakos et al. 2019). Our observed T/C ratio decrease may indicate greater psychological stress in male COMP, characterized by increased fatigue, which was consistent with a case study of a male bodybuilder by Rossow et al. (2013). This increased psychological stress and fatigue in male COMP may partly be explained by achieving very lower body fat and energy intake by the end of the competition preparation (Isola et al. 2023). Supporting this, low energy intake can be particularly challenging for individuals aiming for very low body fat levels through dieting, as observed in bodybuilders during competition preparation (Newton et al. 1993). During the approximately 5-month recovery phase from competition with increased EA, the T/C ratio and fatigue seemed to recover. Monitoring psychological well-being during competition preparation is crucial to mitigate the adverse effects of low energy availability (LEA) and prevent extreme symptoms of relative energy deficiency in sport (RED-S). Supporting mental well-being can reduce mood disturbances, enhance resilience, and improve performance (Angelidi et al. 2024).

We observed the decreases in isometric and concentric lower body strength among male and female COMP during competition preparation. These findings align with previous research on male bodybuilders whose maximal isometric deadlift decreased during competition preparation (Bamman et al. 1993). We previously observed a decrease in upper-body strength among female physique athletes during the competition preparation, while no changes were observed in isometric maximal and explosive lower-body strength during competition preparation (Hulmi et al. 2017). Importantly, isometric and concentric strength returned back to baseline after the recovery phase in the present study and in our earlier study with females (Hulmi et al. 2017). Interestingly, the relationship between the force/CSA ratio was not significantly different from CTRL or between sexes during the competition preparation. Previous studies align with our findings, suggesting that the F/CSA ratio is similar between sexes among similarly trained individuals (Jones et al. 2008), although a firm consensus has not yet been formed. It can be speculated that the stability in the F/CSA ratio, may support lean mass retention and gains sometimes observed during competition preparation (Isola et al. 2023), emphasizing the crucial role of resistance training. Maintaining muscle strength during competition preparation may be an essential goal for preserving FFM and achieving the best possible physique outcomes for the stage (Robinson et al. 2015) as mechanical stimuli are important for muscle hypertrophy (Wackerhage et al. 2019).

Despite the strengths of our study, several limitations must be acknowledged. The follow-up study design does not allow a reliable assessment of causalities. One challenge is that we used MET to estimate EEE, which may not fully capture individual physiological responses. Accurate estimation of EA under real-life conditions is difficult. As noted by (Areta et al. 2021), the quantification of EI and EEE can be inaccurate, affecting our EA estimates. However, the nutritional data is likely to be accurate, considering that physique athletes are known for their strict adherence to diet plans and their expertise in accurately weighing their food portions (Sarin et al. 2022). Additionally, we did not account for potential menstrual cycle variability in female participants. This variability impacts serum estradiol concentrations, complicating the interpretation of hormonal data. Furthermore, in some of the present hormone analyses, the sample size was lower because participants using hormonal contraceptives were excluded, adding to the variability in our hormone analyses for females (ACTH, FSH, estradiol, SHGB). While this variability reflects real-life situations where athletes cannot control the timing of their menstrual cycles during the competition, it nonetheless presents a challenge for data interpretation.

Our results revealed sex-specific physiological responses to energy deficits and established IGF-1 and its binding protein as key, sex-neutral biomarkers for assessing competitive stress. Although many effects of LEA and symptoms of RED-S are observable, our study, along with our previous findings (Isola et al. 2023), indicates that a single 21-week weight loss period does not produce long-term detrimental effects on performance or skeletal health. Additionally, changes induced by competition preparation appear to reverse during recovery when EA is increased pre-competition levels. These insights underscore the need for a holistic approach to training and nutrition that considers physique athletes’ unique physiological and psychological demands. For coaches, athletes, and sports nutritionists, integrating individualized nutrition plans that address specific energy and protein needs and provide mental or psychological health support can mitigate the possible adverse effects of competition preparation, ultimately enhancing athlete performance and well-being.

The findings of this study can inform training and nutritional strategies for physique athletes aiming to optimize performance while minimizing adverse effects. Coaches and sports nutritionists should consider closely monitoring biomarkers such as IGF-1 and IGFBP-3 to gauge physiological stress. Ensuring adequate energy availability and avoiding severe energy deficits can help maintain hormonal balance, muscle mass, and performance. Implementing structured and individualized nutrition plans that focus on energy and protein requirements, alongside mental health support, can mitigate the adverse effects of competition preparation. Additionally, modifying training intensity and volume during competition preparation and incorporating sufficient recovery strategies can help maintain muscle strength and overall well-being.

Future research should focus on longitudinal studies to better understand the long-term effects of competition preparation on hormonal profiles and overall health. It is crucial to investigate the impact of repeated competition cycles on hormonal balance, bone mineral density, muscle mass, and psychological well-being. Studies exploring the effectiveness of various recovery strategies post-competition and their influence on restoring hormonal balance and performance are warranted.

## Data Availability

All data generated or analysed during this study are included in this published article.

## Abbreviations

ACTH: Adrenocorticotropic hormone
ANOVA: Analysis of variance
BM: Body mass
BMD: Bone mineral density
COMP: Competing group
COR: Cortisol
COVID-19: Coronavirus disease 2019
CSA: Cross-sectional area
CTRL: Control group
CV: Coefficient of variation
DXA: Dual-energy X-ray absorptiometry
EA: Energy availability
EEE: Exercise energy expenditure
EI: Energy intake
ELISA: Enzyme-linked immunosorbent assay
FFM: Fat-free mass
FSH: Follicle-stimulating hormone
FT: Free testosterone
HPA: Hypothalamic–pituitary–adrenal
HPG: Hypothalamic–pituitary–gonadal
IBM: International business machines
IGF-1: Insulin-like growth factor 1
IGFBP-3: Insulin-like growth factor binding protein 3
LEA: Low energy availability
LM: Lean mass
MET: Metabolic equivalent
POMS: Profile of mood states
PRE: Pre-competition
POST: Post-competition
RED-S: Relative energy deficiency in sport
SD: Standard deviation
SHBG: Sex hormone-binding globulin
SPSS: Statistical package for the social sciences
T: Total testosterone
VL: Vastus lateralis
WADA: World anti-doping agency
